# Endocrinology in the time of COVID-19

**DOI:** 10.1530/EJE-20-0386

**Published:** 2020-04-17

**Authors:** Wiebke Arlt, Olaf M Dekkers, Juliane Léger, Robert K Semple

**Affiliations:** 1 Institute of Metabolism and Systems Research (IMSR), University of Birmingham, Birmingham, UK; 2 Department of Clinical Epidemiology, Leids Universitair Medisch Centrum, Centrum voor Humane en Klinische Genetica, Leiden, the Netherlands; 3 Department of Paediatric Endocrinology and Diabetes, Head, Reference Center for Rare Endocrine Growth Diseases, Robert Debré University Hospital, Paris, France; 4 Centre for Cardiovascular Science, Queen’s Medical Research Institute, University of Edinburgh, Edinburgh, UK

In what seems like the blink of an eye, many of the routines and certainties of our personal and professional worlds have been systematically dismantled. The global march of the novel coronavirus SARS-CoV-2 and the disease it causes, Covid-19, has played out at exponential pace, and almost no facet of our lives has been left untouched. Many are wrestling with worries for their own health and that of their families, often also riven with concern for their livelihoods. We are denied many of life’s pleasures and leisure activities, prevented from travelling, and starved of gatherings with friends.

Endocrinologists share this personal reality, but have a critical professional role to play as well. Many combine endocrine practice with contribution to acute internal medicine and so can frequently be found on the ‘front line’, directly caring for the most unwell. They must also look to the patients with endocrine or metabolic disease under their speciality care, while dealing with access restrictions to the diagnostic and therapeutic tools we are used to. Endocrinologists must continue to deliver urgent care for their patients while minimising risks of viral transmission, and they must prepare the vulnerable for the worst-case scenario of severe Covid-19. This must be achieved while mitigating collateral harm arising from enforced pausing of less urgent clinical services. Fortunately, children appear to be more mildly affected than adults; however, clinical care delivery for children with endocrine disorders is adjusted in similar ways as in adults due to the overall healthcare crisis.

The clinical practice of endocrinology as we know it depends on quantitative biochemical assessments of hormonal status, often coupled to imaging, generally reliant on sophisticated laboratory and radiological support. Treatments are often delivered by multidisciplinary teams, encompassing nurse specialists, radiologists, surgeons, oncologists, nuclear medicine physicians, ophthalmologists, clinical biochemists, and pathologists among others. We aim for evidence-based practice, with service innovation informed by randomised trials, replicated, corroborated, and meta-analysed. The process is cautious and deliberative, informed by the injunction first to do no harm.

Now the rulebook has been ripped up. The exponentially accelerating speed of the pandemic spread in many countries, and the upheaval of clinical care it has engendered, demands an immediate and rational response. There is no time to ensure that care in times of Covid-19 crisis is strictly evidence-based, and service remodelling must be implemented while facing evolving staff shortages as the pandemic illness also strikes healthcare workers. We must, in short, be adept and nimble at delivering clinical care outside our comfort zone, without resources that we normally take for granted. Consequently, sharing of expert opinion and leadership has rarely been of more importance.

The *European Journal of Endocrinology* is responding to this challenge by providing a platform to share and disseminate such expert advice. We have already started to commission international key opinion leaders involved in front-line endocrine care to deliver clinical management guidance documents in an accessible, bullet point format to facilitate rapid information and adjustment of endocrine service delivery in this time of crisis. In a departure from normal practice, peer review will be highly expedited and open, with reviewers named in the final articles, recognising their critical role in refining recommendations and making them rapidly available. Articles will appear over the next few weeks, with all publication fees waived, and online Open Access publication on the EJE website as soon as the final revised version has been accepted.

We start the first wave of these publication with clinical management guidance on the endocrine and metabolic conditions identified by the EJE Lead Editors as requiring most urgent advice: Adrenal Insufficiency; Cushing’s Syndrome; Pituitary Tumours; Hyponatraemia and Diabetes Insipidus; Hyper- and Hypo-thyroidism; Diabetes in Pregnancy; Restructuring of Diabetes Services in the Time of Crisis; Primary Hyperparathyroidism and Hypercalcaemia; Thyroid Cancer; Adrenal Tumours; and Neuroendocrine Tumours.

These guidance statements are not based on systematic review or meta-analysis (there is no evidence on how to adopt endocrine care in times of a pandemic), but rather on rapid expert consensus; neither are these guidance statements intended to determine absolute standards of medical care. This should, as ever, be carefully tailored to individual circumstances of specific patients, specific hospitals, and specific countries. Nevertheless, in commissioning this series, we have been mindful of the perils of excessive deliberation or over-thinking. As war-based imagery splashes across media coverage of the pandemic, it is perhaps apt to remember the words of the British physicist Robert Watson-Watt, charged with rapidly assembling radar defences at the onset of a world war. In his ‘cult of the imperfect’, channelling an idea articulated by thinkers from Aristotle to Voltaire, he said ‘give them the third best; the second best is too slow and the best never arrives’. This spirit of pragmatic expediency must now animate the endocrine community’s immediate response to help adapt endocrine care to the unforeseen demands of the pandemic crisis.

Focus on short term crisis management does not mean abandoning an evidence-based ideal, however. Hand in hand with forced service re-organisation, many are creatively incorporating research into clinical practice, optimising data and sample acquisition, or even undertaking intervention studies. In due course, these efforts may allow practice to be more solidified around an evidence base, and we shall revisit each of the areas covered now in the time of crisis with more reflective, data-informed analysis once the first peak has passed.

Finally, we have also asked authors to consider where opportunity may arise in disruption. Multidisciplinary, patient-focused care of long-term endocrine conditions lends itself to creative use of remote review and conferencing; both rapid triage of new referrals and regular follow-up of patients with established diagnosis could be undertaken effectively via teleconferencing, preferably by video link, linked to diagnostic assessment in to-be-established peripheral blood letting satellite centres. For many endocrine conditions, self-management by patients is of paramount importance, and a staggered approach, choosing or alternating between face-to-face contact and teleconferencing, would also permit systematic enhancement of patients’ involvement in their own care, following the principles of the 3E framework (Educate, Equip, Empower) and reinforcing their motivation and resilience.

In the response to Covid-19 lies a major opportunity to breach barriers to implementation of more streamlined care pathways exploiting transformative online technologies. Importantly, this challenge forces us to concentrate on the essentials, the ‘clinical bioassay’, that is, the patient himself/herself. This may also facilitate the development of care pathways highly suited to endocrine care delivery in countries with chronically restricted access to the latest and most sophisticated diagnostic and therapeutic modalities. The Chinese word for crisis can be translated either as ‘danger’ or ‘opportunity’. If the endocrine community can grasp this opportunity with determination and ingenuity, then a lasting legacy of good may yet emerge from the dark times in which we currently live. The gauntlet has been thrown down, and the *European Journal of Endocrinology* looks forward to playing its part in the response.

## Declaration of interest

Wiebke Arlt is Editor-in-Chief of *European Journal of Endocrinology*. Olaf Dekkers, Juliane Leger, and Rob Semple are Lead Editors of *European Journal of Endocrinology*. They were not involved in the editorial or review process of this paper, on which they are listed as authors.

## Funding

This editorial did not receive any funding from any funding agency in the public, commercial, or not-for-profit sector.

